# The association of soluble cluster of differentiation 36 with metabolic diseases: A potential biomarker and therapeutic target

**DOI:** 10.1002/pdi3.9

**Published:** 2023-06-12

**Authors:** Yun Li, Yaxi Chen, Xiong Z. Ruan

**Affiliations:** ^1^ Centre for Lipid Research & Chongqing Key Laboratory of Metabolism on Lipid and Glucose The Second Affiliated Hospital Chongqing Medical University Chongqing China; ^2^ John Moorhead Laboratory Centre for Nephrology University College London London UK

**Keywords:** biomarker, cross‐talk, metabolic diseases, soluble CD36, type 2 diabetes mellitus

## Abstract

A cluster of differentiation 36 (CD36), also known as fatty acid translocase, plays an important role in developing and progressing metabolic diseases. Soluble CD36 (sCD36), a circulating form of CD36, is identified in human plasma. Current studies have demonstrated that sCD36 is an early biomarker of type 2 diabetes (T2DM) and atherosclerosis risk and may act as a key molecule in organ cross‐talks directly or indirectly. This review summarizes the cell sources, molecular structure, potential production mechanism, functions, and regulators of sCD36. We highlight the association of sCD36 with hyperlipidemia, metabolic inflammation, T2DM, cardiovascular disease, non‐alcoholic fatty liver disease, diabetic kidney disease, and obesity. These studies suggest that sCD36 could be a useful biomarker for metabolic diseases in children and a potential therapeutic target in preventing metabolic diseases.

## Graphical abstract

In this review, Li et al. summarize the potential production mechanism, molecular structure and functions of sCD36, and highlight the association of sCD36 with metabolic diseases. sCD36 could be a useful biomarker for metabolic diseases and a potential therapeutic target in preventing metabolic diseases.

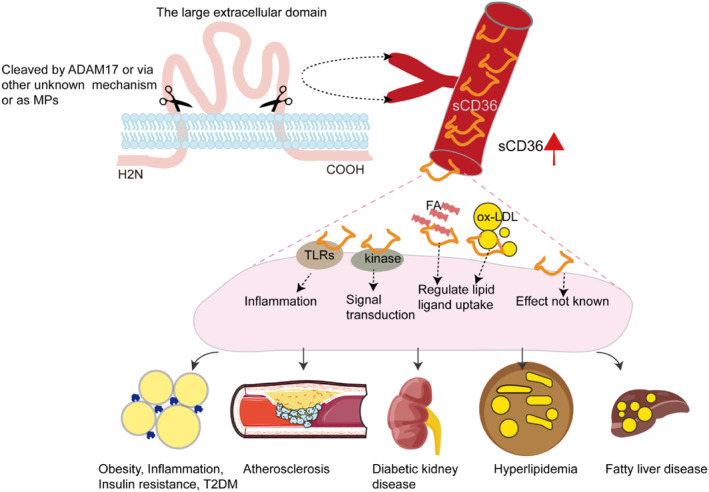

## 1 INTRODUCTION

Metabolic diseases, including obesity, diabetes, cardiometabolic diseases, and non‐alcoholic fatty liver disease (NAFLD) are common worldwide. The etiology of these epidemics is multifactorial and depends on interactions between genetics and environmental factors including diet and physical activity.^1^, ^2^ Inter‐organ cross‐talk has a central role in metabolic syndrome.^3^ Soluble factors in metabolic diseases are capable of mediating molecular cross‐talks between organs directly or indirectly.^4^, ^5^

A cluster of differentiation 36 (CD36), also known as fatty acid translocase (FAT), is a transmembrane glycoprotein that contains several posttranslational modification sites. It is widely expressed in adipocytes, endotheliocytes, skeletal muscle cells, macrophages, and platelets. It binds to diverse ligands, including apoptotic cells, thrombospondin‐1, fatty acids, and oxidized low‐density lipoprotein.^6^ CD36 participates in NAFLD, atherosclerosis, chronic kidney disease, diabetes mellitus, immunity, and cancer.^7^, ^8^, ^9^, ^10^ Soluble CD36 (sCD36) is a circulating form of CD36 and was identified in human plasma.^11^ Current studies show that sCD36 may be a new potential biomarker in diabetes mellitus and is closely related to metabolic diseases such as type 2 diabetes mellitus (T2DM), insulin resistance, atherosclerosis, fatty liver, inflammation, and obesity.^11^, ^12^, ^13^, ^14^, ^15^ In the last decade, however, few reviews have systematically evaluated the published evidence on the relationship of sCD36 with metabolic diseases. In this paper, we review the association of sCD36 with metabolic diseases and its underlying pathophysiological mechanisms by searching PubMed and Web of Science databases. Clarifying the role of sCD36 in metabolic disorders and associated complications can provide a theoretical basis for the early diagnosis of diseases and exploring therapeutic strategies.

## 2 THE SOURCE AND STRUCTURE OF sCD36

### 2.1 Cell source of sCD36

CD36 is widely expressed on the surface of adipocytes, platelets, monocytes, macrophages, and endotheliocytes.^16^ Until now, the source of sCD36 has not been clearly determined. In 2006, Handberg et al. discovered the existence and possible sources of sCD36. CD36 in the circulation may be an unbound protein or exist as peptide, or may exist in particles shed from platelets, monocytes/macrophages, or adipocytes after being triggered by various stimuli.^11^ Handberg then concluded that sCD36 reflects the expression of CD36 in some tissues associated with metabolic syndrome, especially in monocytes and macrophages.^17^ Macrophage infiltration and low‐grade inflammation in abdominal obesity may lead to dyslipidemia, lipoprotein peroxidation, and liver steatosis, and these factors promote the development of atherosclerosis and induce CD36 in the vascular wall. Metabolic inflammation and insulin resistance stimulate CD36 expression of monocytes and macrophages in adipose tissue, the liver, and the artery, increasing plasma sCD36.^17^

CD36 is involved in ectopic lipid accumulation. sCD36 in NAFLD patients increased with insulin resistance, the level of intrahepatic lipid and dyslipidemia.^13^, ^17^ The weak correlation with obesity markers and the correlation with hepatic CD36 mRNA expression suggested that hepatic CD36 might contribute to the sCD36 circulation pool.^18^ Liver‐derived soluble factors play an important role in the pathogenesis of vascular disease associated with liver cirrhosis. If sCD36 derives from hepatocytes, sCD36 may contribute to the extrahepatic complications related to NAFLD including atherosclerosis.

CD36 is one of the major glycoproteins of platelets and known as GPIV. Alkhatatbeh et al. found that CD36 was not in the components of the separated lipoproteins, suggesting that CD36 was neither a lipoprotein nor a proteolytic fragment. Instead, it was associated with microparticle fragments in plasma, suggesting sCD36 was a product of circulating microparticles.^19^ These microparticles were mainly derived from platelets. In vitro, activated platelet analysis also showed that CD36 was secreted in the form of microparticle. More specific studies on the cell sources of elevated sCD36 were required. Further, they found that CD36‐positive microparticles (CD36+ MPs) were significantly increased in obese patients with T2DM, and these CD36+ MPs were mainly derived from red blood cells, In contrast, the main source of CD36+ MPs in non‐T2DM patients was endothelial cells. In the whole cohort, the plasma CD36 protein was positively correlated with the CD36+ MPs detected by flow cytometry. Multivariate analysis showed that the plasma CD36+ MP level was a better biomarker of diabetes than the CD36 protein concentration.^20^ They also indicated that sCD36 was not a product of CD36 proteolysis or other isoforms. However, one study showed that the level of sCD36 in diabetes patients who did not take aspirin was significantly higher than that in healthy subjects. sCD36 was significantly lower in diabetes patients who received aspirin treatment than in those who did not. Downregulation of sCD36 by low‐dose aspirin was incomplete, indicating that sCD36 may come from tissues other than platelets.^21^

In hyperlipidemic conditions, multiple cell types contribute to circulating CD36 generation, with a particularly strong contribution from endothelial cells. Oxidized phospholipids, ligands for CD36, which are known to accumulate in circulation in hyperlipidemia, induce a robust release of CD36 from several cell types. In vivo, CD36 releases into the circulation of mice in response to tail‐vein injection of oxidized phospholipids.^22^

In addition, the expression of CD36 on the surface of myeloid cells in the blood, brain, and bone marrow of adult diabetic (db/db) mice which carry a mutation in the gene encoding leptin receptor, was significantly decreased when compared with normoglycemic control heterozygous db/+ mice. The researchers believe that this is related to the increase of plasma sCD36.^23^ Shiju et al. found sCD36 in urine first and speculated that the circulating microparticles released by apoptotic proximal tubular epithelial cells were CD36 positive. They believed that sCD36 fell off from the cells as microparticles and exists in the circulation as peptide molecules when triggered by stimuli and might even be released during apoptosis.^24^ We summarized the possible cell sources of sCD36 in Table 1.

**TABLE 1:** Cell sources of soluble CD36 (sCD36).

Cells	Form in the plasma	Ref.
Monocytes	An unbound protein or a peptide	[11, 17]
Macrophages	An unbound protein or a peptide	[11, 17]
Hepatocytes	An unbound protein or a peptide	[13, 17]
Platelets	Microparticle component	[19, 20, 21]
Endothelial cells	An unbound protein or a peptide	[22]
Cells in blood, brain, and bone marrow	An unbound protein or a peptide	[23]
Apoptotic proximal tubular epithelial cells	A peptide	[24]

Abbreviations: CD36, cluster of differentiation 36; sCD36, soluble CD36.

### 2.2 Structure of sCD36

**FIGURE 1:**
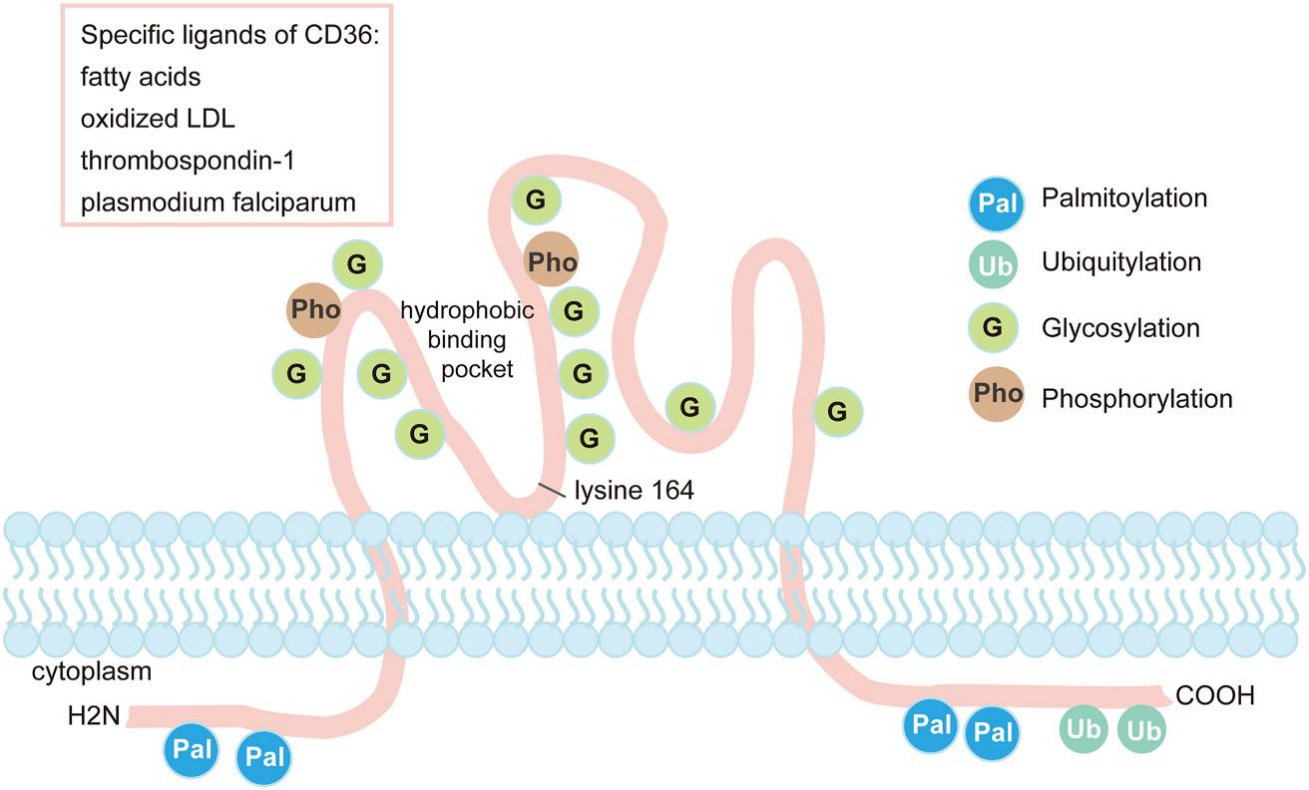
CD36 structure and post‐translational modifications. CD36 comprised of 472 amino acids and folded into a single peptide chain, with two transmembrane domains and two small cytoplasmic tails. CD36 contains several post‐translational modifications, including four palmitoylation sites, two phosphorylation sites, two ubiquitylation sites, and ten N‐linked glycosylation sites. The hydrophobic CD36 binding pocket was required for lipid transport. CD36, cluster of differentiation 36.

CD36 is a transmembrane glycoprotein receptor, comprising of 472 amino acids and folded into a single peptide chain, with two transmembrane domains and two small cytoplasmic tails.^16^, ^25^, ^26^ CD36 contains several post‐translational modifications, including four palmitoylation sites, two phosphorylation sites, two ubiquitylation sites, and ten N‐linked glycosylation sites. The hydrophobic CD36 binding pocket was required for lipid transport (Figure 1). sCD36 may be a free form of the extracellular domain of CD36 that is shed into the blood after proteolysis.^27^, ^28^ Wilson et al. constructed the soluble CD36 extracellular domain, which can bind to the negatively charged diacylglycerol ligand and act as a co‐receptor of toll‐like receptors (TLR) 2 to activate inflammation.^27^ Wang et al. expressed the extracellular domain of human CD36 (gly30‐asn439) in *Escherichia coli*, which can specifically bind with oxidized low‐density lipoprotein (ox‐LDL) and effectively inhibit the uptake of ox‐LDL by mouse macrophage RAW 264.7 cells.^28^ CD36 on platelets was initially described as a proteolytic enzyme‐resistant glycoprotein. Thus, researchers inferred that sCD36 may be the extracellular domain of CD36. Proteolytic cleavage is a mechanism capable of releasing soluble apoptotic cell receptors.^29^ A disintegrin and metalloprotease protein‐17 (ADAM17) is a ubiquitously expressed membrane‐bound enzyme. CD36 on the cell surface was increased in macrophages of ADAM17 null mice, while sCD36 was decreased.^30^, ^31^ This suggests that ADAM17 is involved in the proteolytic cleavage of CD36. The ADAM family hydrolyzes molecules expressed on the cell surface, thereby controlling the immune response. For example, ADAM10 and ADAM17 participate in the cleavage of pattern recognition receptors, including the clearance receptors CD163, CD36, chemokine (C‐X‐C motif) ligand 16, and TLR2.^32^ However, there was no significant change in CD36 on the surface of monocytes after ADAM17 knockdown. Moreover, no sCD36 was detected. Further studies are required to clarify the mechanism by which sCD36 is formed.

## 3 THE BIOLOGY FUNCTION OF sCD36

CD36 has important roles in metabolic diseases, including atherosclerosis,^33^, ^34^ NAFLD,^35^, ^36^, ^37^ obesity,^38^ chronic metabolic inflammation,^39^ and diabetes mellitus,^40^, ^41^, ^42^, ^43^ as well as hyperlipidemia,^44^ through binding with multiple ligands. Wilson found that the extracellular domain of sCD36 is bound to the negatively charged diacylglycerol ligand and acts as a co‐receptor of TLR2.^27^ The pro‐inflammatory effect of sCD36 may be related to the activation of the immune response against pathogens and the progression of chronic diseases. This suggests that elevated sCD36 in T2DM patients may accelerate vascular complications of T2DM, such as atherosclerosis, by increasing the pro‐inflammatory response to diacylglycerol ligands. His6‐tagged sCD36 containing the extracellular domain of human CD36 (Gly30‐Asn439) bonded ox‐LDL specifically and effectively inhibited ox‐LDL uptake by RAW 264.7 cells.^28^ Three active compounds, Danshen sodium, Rosmarinic acid, and Salvianolic acid B, had antagonistic effects on the binding of sCD36‐oxLDL and further inhibited ox‐LDL uptake in RAW 264.7 cells.^28^ This study provides an idea for identifying bioactive components targeting atherosclerosis. Long chain omega‐3 polyunsaturated fatty acids have cardioprotective effects. However, circulating the CD36+ MPs is not altered by docosahexaenoic acid or eicosapentaenoic acid supplementation.^45^ The cardioprotective effects of docosahexaenoic acid and eicosapentaenoic acid did not act through a CD36+ MP mechanism. HCV infection in vitro increased CD36 expression in either surface or soluble form.^46^ CD36, CD163, and low‐density lipoprotein receptor‐related protein 1 (LRP1) can bind with circulating red blood cells, haptoglobin‐hemoglobin complexes, and heme‐hemoglobin complexes, respectively. Reducing free circulating red blood cells and the above complexes reduce neuro‐inflammation, oxidative stress, and cell death, thus improving the prognosis of hemorrhagic stroke.^47^ The soluble forms of these receptors, namely sCD36, sCD163, and sLRP1, may become decoy receptors as binding proteins for these molecules.^48^ Currently, only a small amount of research has revealed the biological function of sCD36. Given that the CD36 protein has multiple ligand‐binding regions, we speculate that sCD36 may also bind with corresponding ligands to play roles in metabolic diseases and even be involved in the cross‐talk between organs. We summarized the roles of CD36 and sCD36 in Table 2. Identifying the structure of sCD36 and constructing a plasmid containing sCD36 may be beneficial for analyzing the function of the circulating sCD36.

**TABLE 2:** Roles of CD36 and sCD36 in metabolic diseases.

Metabolic diseases	CD36	sCD36
Diabetes	Optimizes lymphatic lipid transport^41^	Still far from clear
	Regulates glucose metabolism^42^	
	Promotes inflammation^43^	
CVD	Mediates signal transduction^26^	Promotes inflammation and accelerates vascular complications of T2DM^27^
	Mediates ox‐LDL uptake and disturbs lipid metabolism^26^	Bonds ox‐LDL specifically and effectively inhibits ox‐LDL uptake by RAW 264.7 cells^28^
	Regulates immune response^26^	
NAFLD	Increases FFA uptake and drives hepatosteatosis onset^36^	Still far from clear
	Promotes inflammation in the progression of NASH^37^	
Inflammation	Drives chronic inflammation^39^	A co‐receptor of TLR2^27^
		Reduce neuro‐inflammation^47^
The kidney disease	Regulates lipid metabolism and inflammation^16^	Still far from clear
Obesity	Facilitates FFA uptake^38^	Still far from clear
Hyperlipidemia	Channels lipids into BAT^44^	Still far from clear

Abbreviations: BAT, brown adipose tissue; CD36, cluster of differentiation 36; CVD, cardiovascular disease; FFA, free fatty acids; NAFLD, non‐alcoholic fatty liver disease; NASH, non‐alcoholic steatohepatitis; ox‐LDL, oxidized low density lipoprotein; sCD36, soluble CD36; T2DM, type 2 diabetes mellitus; TLR2, toll like receptor 2.

## 4 THE REGULATOR OF THE sCD36

Green tea polyphenols reduced the risk of coronary artery disease.^49^, ^50^ High glucose significantly induced the overproduction of sCD36, while the consumption of green tea polyphenols inhibited the increase of sCD36 caused by fructose intake.^51^ In addition, sCD36 was induced by a high‐fat diet, and cinnamon extract reduced sCD36 by 37%.^52^ Hussain's team found that the increased expression of the rhythm gene clock led to a decrease in the expression of CD36 when exposed to ox‐LDL.^53^ Sylvain Doré speculated that sCD36 might also exhibit different expressions due to circadian atherosclerosis.^48^ Analysis of the data available led us to conclude that targeting sCD36 would be a potential strategy to promote/inhibit the crosstalk between the organs and even reduce complications.

## 5 sCD36 IS ASSOCIATED WITH METABOLIC DISEASES

### 5.1 sCD36 is elevated in T2DM

Globally, about 1 in 11 adults worldwide now have diabetes mellitus, 90% of whom are T2DM. T2DM is increasing rapidly with alarming trends in children and young adults (up to the age of 40 years). Early detection and proactive management are crucial for preventing and mitigating microvascular and macrovascular complications and mortality burden.^54^, ^55^ Oral glucose tolerance tests and glycated hemoglobin (HbA1c) are widely used for the diagnosis of diabetes.^56^ In 2010, the American Diabetes Association included HbA1c ≥6.5% in the revised criteria for the diagnosis of diabetes. However, the debate as to whether HbA1c should be used to diagnose diabetes is far from being settled, and there are still unanswered questions regarding the cut‐off value of HbA1c for diabetes diagnosis in different populations and ethnicities.^57^ New clinical biomarkers may be needed for the diagnosis of diabetes mellitus.

The proteomic knowledge has provided new insight into the pathophysiology of T2DM and its complications. In 2006, Handberg et al. first demonstrated the existence of a circulating form of CD36 in plasma by immunopurification and Western blotting and established an ELISA assay to detect sCD36 in plasma.^11^ CD36 is an important component of the pathogenesis of diabetes mellitus and its complications due to the effects mediated by lipid transport, alterations in insulin response, and glucose metabolism.^40^, ^41^, ^42^, ^43^, ^58^ Handberg et al. found that sCD36 was markedly elevated in T2DM patients compared with both lean (5‐fold) and obese (2‐ to 3‐fold) control subjects, and sCD36 was highly related to risk factors of accelerated atherosclerosis in T2DM such as insulin‐stimulated glucose disposal, fasting plasma glucose, fasting insulin, and body mass index (BMI). This study concluded that sCD36 might be a marker of metabolic syndrome and a potential surrogate marker of atherosclerosis. On the basis of this important observation, the team further demonstrated that sCD36 and risk markers of insulin resistance and atherosclerosis were increased in patients with polycystic ovary syndrome and significantly decreased during pioglitazone treatment, further supporting the association between sCD36 and insulin resistance in patients with polycystic ovary syndrome.^59^ sCD36 clusters with important markers of insulin resistance and metabolic syndrome that are key predictors of T2DM. High sCD36 is associated with increased T2DM risk independent of age, sex, and obesity.^60^ In another cross‐sectional study, sCD36 in plasma was positively associated with fasting insulin, triglycerides, low‐density lipoprotein (LDL), systolic blood pressure, BMI, and waist circumference but negatively correlated with high‐density lipoprotein in clinically healthy Caucasians, suggesting that sCD36 was significantly correlated with insulin resistance.^61^ sCD36 was negatively correlated with insulin sensitivity and positively correlated with fasting blood glucose, fasting triglycerides, fat‐free body weight, and blood platelet count in subjects with glucose intolerance.^14^ Increasing carotid intima‐media thickness has been implicated in the development of cognitive decline and dementia and was inversely correlated with the cognitive function of T2DM patients.^62^ Zhou et al.^63^ found that the higher sCD36 level in T2DM patients was associated with carotid intima‐media thickness. However, no cross‐sectional association between sCD36 and the Montreal Cognitive Assessment scores was found in this study. sCD36 could not be used as a risk marker for cognitive impairment in T2DM patients. In these observational studies, sCD36 is closely related to insulin resistance. However, it is not clear whether sCD36 can be used as a surrogate indicator to predict the risk of diabetes. In 2017, researchers found that sCD36 index was independently related to the risk of T2DM, and the correlation was better than the triglyceride‐glucose index,^64^ suggesting that sCD36 may be an effective surrogate indicator for the risk of diabetes. Similar to blood glucose, sCD36 levels were higher in the T2DM patients compared with healthy volunteers in a comparative study, and elevated sCD36 increased the T2DM risk,^65^ suggesting that sCD36 may be a potential biomarker for T2DM. However, there was no correlation between sCD36 level and homeostatic model assessment of insulin resistance (HOMA‐IR) value, blood insulin and triacylglycerol levels, waist circumference, dietary fatty acid pattern, and food preferences in the T2DM patients. Compared with healthy individuals, serum sCD36 in T2DM patients with hypertension increased significantly, and sCD36 correlated significantly with serum PPAR‐γ. T2DM patients with elevated sCD36 may have early subclinical atherosclerosis.^66^ However, another study showed that sCD36 was weakly associated with T2DM, and not with T1DM. Circulating plasma sCD36 might not be a biomarker of T1DM or T2DM, but it did not wholly exclude the possibility that sCD36 may be a biomarker of cardiovascular events in diabetes and other populations.^67^ These studies suggest that sCD36 may be responsible for macrovascular complications in patients with diabetes and contribute to the crosstalk networks between T2DM and atherosclerosis. In addition, sCD36 is also correlated with urea, creatinine, and estimated glomerular filtration rate, suggesting a role of sCD36 in diabetic kidney disease and microvascular complications of diabetes.

Recognizing sex differences in medicine is one step toward personalized medicine.^68^ The effect of sex on sCD36 concentration was controversial. In 2018, M.E. RAC' et al. observed that the female sex was a significant independent predictor of a higher sCD36 concentration.^69^ However, Chmielewski and colleagues found that the serum sCD36 concentration in Caucasian patients with chronic kidney disease was independent of sex.^70^ Additionally, M.E. RAC' et al. showed that sCD36 concentration in the serum of patients with coronary heart disease was negatively correlated with uric acid and insulin concentration, HOMA‐IR ratio, and BMI but positively correlated with high‐density lipoprotein and apolipoprotein A1 concentration, suggesting a protective effect of higher sCD36 on metabolic syndrome components.^69^ In general, more evidence on the relationship between sCD36 and diabetes may be required in different physiological and pathophysiological states.

### 5.2 sCD36 may be involved in the development of cardiovascular diseases

Cardiovascular diseases (CVD) remain one of the leading causes of morbidity and mortality worldwide and are the top cause of death in China.^71^, ^72^ Diagnosing CVD early and accurately is critical to refer high‐risk individuals for intensive lifestyle modification.^73^ Remarkable advances have been made in basic and clinical research fields, such as hair cortisol and trimethylamine N‐oxide.^74^, ^75^ CD36 is closely associated with the progression of cardiovascular diseases because CD36 is decreased in pathological cardiac hypertrophy caused by ischemia–reperfusion and pressure overload and increased in diabetic cardiomyopathy and atherosclerosis.^26^, ^34^ In 2008, Handberg et al. first demonstrated that sCD36 was markedly elevated in the plasma of patients with symptomatic atherosclerotic carotid plaques and was related to plaque instability, suggesting that sCD36 may be a marker of plaque instability and symptomatic carotid atherosclerosis, and at least in part, it may be due to the release of CD36 from foam cells in atherosclerotic lesions to the circulation.^12^ In the non‐diabetic cross‐sectional study by Handberg in 2012, sCD36 was significantly correlated with insulin resistance index, carotid atherosclerosis, and fatty liver,^61^ which provided more evidence of the role of sCD36 in atherosclerosis. CD36 has an important role in endothelial cells, smooth muscle cells, monocytes, macrophages, and platelets in the development of atherosclerosis.^26^ This suggested that sCD36 may be a reliable biomarker for the diagnosis of atherosclerosis. Cardiovascular disease, T2DM, metabolic syndrome, obesity, and plaque instability are known precursors of stroke. Increased pro‐inflammatory proteins in the circulation may increase stroke incidence and significantly aggravate stroke progression. sCD36 may be a potential therapeutic target for preventing or treating stroke.^48^ The concentration of sCD36 in patients with ST‐segment elevation myocardial infarction is higher than that in patients with unstable angina, indicating that the concentration of sCD36 may be related to the clinical spectrum of acute coronary syndrome patients.^76^

However, M.E. RAC' et al. showed that sCD36 was negatively associated with apolipoprotein B/apolipoprotein A1 ratio, BMI, waist‐hip ratio, systolic blood pressure, left ventricular end‐diastolic diameter and volume, left atrium diameter, right ventricular end‐diastolic diameter and was positively correlated with high‐density lipoprotein cholesterol and apolipoprotein A1 concentration,^69^, ^77^ highlighting a protective role of higher sCD36 in patients with coronary artery disease. Higher sCD36 concentration is also associated with a lower risk of left ventricular hypertrophy, but on the other hand, is a potential risk factor for impaired left ventricular diastolic function.^77^ Additionally, M. J. Alkhatatbeh et al. found that sCD36 concentration was relatively low in middle‐aged subjects, and there was no significant difference in sCD36 between subjects with obesity, hyperglycemia, dyslipidemia, hypertension or CVD, and those without these abnormalities.^78^ sCD36 was also unrelated to BMI, age, glucose, lipid profile, blood counts, and serum electrolytes,^78^ indicating that sCD36 concentration was not associated with cardiovascular risk factors in middle‐aged subjects. This is inconsistent with a previous study that sCD36 and ox‐LDL were associated with cardiovascular risk factors, including obesity and hypertriglyceridemia in young subjects and may be potential early markers of cardiovascular disease.^79^ Another study also pointed out that high sCD36 was observed only in female patients with a low ankle‐brachial index, and sCD36 did not have a strong correlation with the radiological parameters of atherosclerosis nor did it prove the harmful or beneficial effects of sCD36 on atherosclerosis.^80^ The sCD36 concentration was not affected by excessive fetal growth in fetal macrosomia. Nonetheless, the cord blood pentraxin‐3 and respective sCD36 concentrations in the macrosomic group were positively correlated, indicating an inflammatory pathway, linking extreme fetal growth with the predisposition to metabolic syndrome and cardiovascular pathology later in life.^81^ A multivariate model showed that sCD36 was not correlated with subclinical carotid atherosclerosis in the three study groups of T1DM, T2DM, and non‐diabetes patients.^82^ In 2021, Mauricio et al. evaluated the relationship between circulating CD5 molecule‐like or sCD36 and the risk of cardiovascular events in chronic kidney disease. They showed that sCD36 was not associated with adverse cardiovascular outcomes or mortality.^83^

CD36 plays an important role in the development of atherosclerosis. Targeting CD36 is a potential strategy for atherosclerosis. sCD36 may be a potential biomarker for the diagnosis of atherosclerosis and involved in the crosstalk between the heart and other organs. However, for the controversial or opposed results in current studies, different analyzed populations and a lack of reliable methods to determine sCD36 may be responsible. The role of sCD36 in cardiovascular diseases deserves further investigation.

### 5.3 The elevation of sCD36 is consistent with hepatic steatosis in NAFLD

NAFLD is the most common cause of chronic liver disease worldwide. One of the crucial events involved in NAFLD progression is the lipotoxicity resulting from an excessive fatty acid influx to hepatocytes.^84^, ^85^ CD36 increases the uptake of free fatty acids in the liver, and it drives hepatosteatosis onset and might contribute to its progression to non‐alcoholic steatohepatitis.^37^, ^86^ Interestingly, circulating sCD36 is also abnormally elevated in NAFLD patients. In 2009, Fernández‐Real and Handberg found that sCD36, in a male cohort, was positively correlated with alanine aminotransferase, aspartate aminotransferase, and gamma‐glutamyl transferase in subjects with impaired glucose tolerance but not in individuals with normal glucose tolerance,^87^ suggesting a correlation between alanine aminotransferase and sCD36 and that circulating sCD36 may be a new marker of liver injury in subjects with altered glucose tolerance. Another study on sCD36 and NAFLD indicated that sCD36 level correlated with the histological grade of steatosis and was an independent factor associated with advanced steatosis in NAFLD patients.^13^ sCD36 was correlated with intrahepatic lipid levels in NAFLD patients. sCD36 in NAFLD patients was higher than those in the control group. sCD36 was also correlated with alanine aminotransferase, HOMA‐IR, high‐density lipoprotein, and triglyceride. Multiple regression analysis showed that intrahepatic lipids and plasma triglyceride were independent predictors of sCD36. sCD36 increased with elevated intrahepatic lipid, insulin resistance and dyslipidemia.^18^ sCD36 is considered as a potential biomarker of the steatosis severity in fatty liver disease. It may be beneficial for NAFLD epidemiology, non‐invasive diagnosis, prediction of treatment outcome, and prognosis in the future.^36^, ^61^ However, a study found no significant differences between sCD36 concentration in patients with obesity and NAFLD and non‐hepatopathy patients with obesity.^88^ And they also did not find a significant difference between sCD36 concentration in children with obesity compared to the control group and between mild and advanced steatosis. Hepatic CD36 expression was abnormally upregulated in NAFLD mice and patients and promoted the development of NAFLD. The role of sCD36 in fatty liver diseases is worth an in‐depth study.

### 5.4 sCD36 is positively correlated with chronic metabolic inflammation

CD36 signaling in macrophages links dysregulated fatty acid metabolism to oxidative stress from the mitochondria, which drives chronic inflammation. Targeting to CD36 and its downstream effectors may serve as potential new strategies against chronic inflammatory diseases.^39^ In addition to the correlation between sCD36 and T2DM or cardiovascular disease, Fernández‐Real and Handberg also found that sCD36 was positively correlated with interleukin 6. This study first proposed the correlation between inflammation and sCD36.^14^ Moreover, in high‐fructose‐fed rats, sCD36 was correlated with tumor necrosis factor‐alpha and interleukin 6.^51^ Additionally, Handberg proposed a model in which low‐level inflammation may lead to increased sCD36.^17^ CD36 has been shown to cooperate with TLR2 and can mediate signal transduction and inflammation. Circulating sCD36 may also mediate signal transduction or immune responses by interacting with pattern recognition receptors at the cell surface.

### 5.5 sCD36 may be responsible for diabetic kidney disease

Ruan et al. summarized that renal CD36 expression is upregulated by hyperlipidemia and hyperglycaemia. Patients with chronic kidney disease, particularly those with diabetic nephropathy, show increased CD36 expression.^16^ Renal CD36 is involved in lipid accumulation, inflammation, and kidney fibrosis through activation of the pattern recognition receptors and several signaling pathways.^16^, ^89^, ^90^ In 2010, Michal Chmielewski et al. first reported the correlation between sCD36 and kidney disease.^70^ The serum sCD36 concentrations of chronic kidney disease stage 5 patients before dialysis were significantly higher than that in healthy individuals. The use of HMG‐CoA reductase inhibitors (statins) reduced the sCD36. The sCD36 concentration can predict the cardiovascular mortality of chronic kidney disease stage 5 patients. In 2015, Viswanathan and colleagues found sCD36 in the urine sample.^24^ sCD36 in plasma and urine of diabetes patients with microalbuminuria and macroalbuminuria were significantly increased. sCD36 was positively correlated with renal markers such as urea, creatinine, and estimated glomerular filtration rate, which confirmed the correlation between sCD36 and renal damage in patients with diabetes. Microalbuminuria, a biomarker used clinically for nephropathy, was strongly and positively correlated with urine and plasma sCD36. sCD36 may be responsible for the occurrence of microvascular complications of T2DM and may be available for auxiliary diagnosis for diabetic kidney disease. More evidence is required to clarify the role of sCD36 in diabetic nephropathy and other chronic kidney diseases.

### 5.6 sCD36 may be an early indicator of long‐term health risks related to obesity and complications

CD36 facilitates fatty acid uptake and promotes obesity.^38^ CD36 deficiency protects against diet‐induced obesity, intramuscular lipid deposition, and oxidative stress.^91^ sCD36 is also related to obesity. sCD36 levels were higher in obese subjects than normal‐weight controls.^79^ Obese subjects had a 5.8 times higher risk of sCD36 in the third tertile than normal‐weight controls. However, in another study, BMI was negatively correlated with the sCD36 level in young women.^92^ The association of sCD36 with BMI needs to be studied in larger populations. The effect of sCD36 may increase BMI and promote the development of obesity and associated metabolic diseases if sCD36 still has domains where lipid ligands bind. In general, sCD36 may be an early indicator of long‐term health risks related to obesity and obesity‐related complications.

### 5.7 sCD36 may be associated with hyperlipidemia

Hyperlipidemia is a condition characterized by oxidative stress and low‐grade inflammation. CD36 facilitates the uptake of triglycerides into brown adipose tissue in response to short‐term cold exposure and contributes to the accelerated plasma clearance of triglycerides in the pathophysiological setting of hyperlipidemia.^44^ sCD36 is increased in hyperlipidemic apolipoprotein E knockout mice and low‐density lipoprotein receptor‐deficient mice. This finding documents sCD36 in hyperlipidemia and identifies a link between sCD36 and oxidized phospholipids generated under oxidative stress and low‐grade inflammation associated with hyperlipidemia.^22^

### 5.8 sCD36 may be a suitable novel risk marker at the origin of metabolic diseases in obese children

CD36 plays an important role in metabolic disease in children. CD36 is an adipocyte progenitor marker, and its expression is associated with adipose tissue function in children.^93^ Single‐nucleotide polymorphisms in CD36 are associated with macular pigment among children.^94^ Until now, there is limited literature on the relevance of sCD36 for children. Handberg et al. conducted a 10‐week lifestyle intervention on 113 overweight children and found that weight loss can reduce the sCD36.^15^ The reduction of sCD36 after weight loss was significantly in correspondence with improved insulin sensitivity, dyslipidemia, and liver fat estimated by ultrasonography in obese children. In a survey of vascular characteristics and phenotypes of CVD in obese children and adolescents, sCD36 levels in obese children with metabolic syndrome were significantly higher than those in their peers without abnormalities. Still, there was no difference among adolescents.^95^ However, one study consisting of 50 children with obesity aged 8–17.5 years showed no significant difference in sCD36 between children with obesity and the control group.^88^ sCD36 level in patients with obesity and NAFLD was consistent with that in non‐hepathopathy patients with obesity.^88^ Further studies on a larger pediatric population are needed to determine the controversial role of sCD36 in metabolic diseases in children.

## 6 CONCLUSION

Clinical studies have shown that plasma sCD36 is associated with metabolic diseases in adults and children. sCD36 may be a potential biomarker of T2DM, atherosclerosis, metabolic inflammation, and other pathological processes. It is a good prospect for the early diagnosis of metabolic diseases in the clinic. Moreover, circulating sCD36 released from cells or organs may be involved in the cross‐talk in metabolic diseases. sCD36 may be a potential therapeutic target. Until now, most of the current studies are retrospective case‐control studies. Experiments and more evidence are needed to clarify the structure of sCD36. A vector containing sCD36 would contribute to determining the function of sCD36 and the underlying mechanisms. More prospective cohort studies would be significantly important for elucidating the role of sCD36 in T2DM or other metabolic diseases. These would be beneficial for preventing metabolic disease‐related complications and exploring therapeutic strategies targeting sCD36. However, the detection of specific soluble proteins was affected by high abundant proteins in the plasma. It is necessary to improve the sensitivity of sCD36 detection. The quality of commercially available sCD36 ELISA kits and anti‐CD36 antibodies varies. A standardized simple sCD36 ELISA that could be performed in any basic laboratory would be more favorable. In addition, the incidence of metabolic diseases in children and adolescents is increasing. The role of sCD36 in children needs to be clarified in a larger pediatric population which would be a specific research question in the future.

## AUTHOR CONTRIBUTIONS

Yun Li and Xiong Z. Ruan: conceptualization; Yun Li: writing – original draft; Yun Li, Yaxi Chen and Xiong Z. Ruan: writing – review & editing; Xiong Z. Ruan: supervision; Yun Li and Xiong Z. Ruan: funding acquisition.

## CONFLICT OF INTEREST STATEMENT

Xiong Z. Ruan is the member of *Pediatric Discovery* Editorial Board. He was excluded from all editorial decision‐making related to the acceptance of this article for publication. The remaining authors declare no conflict of interest.

## ETHICS STATEMENT

This article does not contain any studies with human participants.

## Data Availability

Data sharing not applicable to this article as no datasets were generated or analyzed during the current study.
